# Supplementary material for: Retrosynthetic planning with experience-guided Monte Carlo tree search
Source: Commun Chem. 2023 Jun 10;6:120. doi: 10.1038/s42004-023-00911-8 (PMC10257190; doi:10.1038/s42004-023-00911-8)
Supplement: Supplementary file 2 — Supplementary Information.pdf [file 42004_2023_911_MOESM2_ESM.pdf]

Supplementary Information

Siqi Hong[1], Hankz Hankui Zhuo[1*], Kebing Jin[1], Guang Shao[2], Zhanwen Zhou[1]

[1*]School of Computer Science and Engineering, Sun Yat-Sen University, East Outer Ring Road, Guangzhou, 510006, Guangdong, China.
[2]School of Chemistry, Sun Yat-Sen University, East Outer Ring Road, Guangzhou, 510006, Guangdong, China.

*Corresponding author(s). E-mail(s): zhuohank@mail.sysu.edu.cn;
Contributing authors: hongsq@mail2.sysu.edu.cn; jinkb@mail2.sysu.edu.cn; shaog@mail.sysu.edu.cn; zhouzhw9@mail.sysu.edu.cn;

1 Supplementary Table 1

2 Supplementary Figure 1

Supplementary Table 1. 30 testing molecules used in the experiment comparing the generated routes given by computer approaches EG-MCTS. It shows the CAS Number of each molecule. If the corresponding synthetic route is reported in the patent, the table also shows the Patent Number. Otherwise it shows the journal name. We also provide the computer-readable forms of the published routes of these thirty molecules in the code.

Index	CAS Number	Published Route
1	1448441-60-8	WO 2013107283
2	895520-52-2	WO 2006069153
3	1100216-25-8	WO 2009009411
4	1392842-01-1	WO 2012106472
5	1443043-01-3	US 20130150407
6	1173981-96-8	US 20090186879
7	1173980-10-3	US 20090186879
8	866920-26-5	WO 2005097786
9	1352087-71-8	FR 2960876
10	1448441-53-9	International Journal of Cancer
11	749922-13-2	WO 2006028451
12	1451094-21-5	US 20130225588
13	1100217-13-7	WO 2009009411
14	1100216-27-0	WO 2009009411
15	1617516-73-0	US 20140194476
16	1173979-95-7	US 20090186879
17	1203552-27-5	WO 2010000773 and WO 2013079708
18	1040247-00-4	WO 2008089459
19	1173978-72-7	Bioorganic Medicinal Chemistry
20	1173979-96-8	US 20090186879
21	1392843-72-9	WO 2012106472
22	769169-77-9	US 20040198778
23	1451094-35-1	US 20130225588
24	1498291-86-3	WO 2013180265
25	1801756-11-5	WO 2013107283
26	1392841-71-2	WO 2012106472
27	1873306-29-6	WO 2016016368
28	345963-30-6	WO 2002018361
29	1392841-74-5	WO 2012106472
30	1246199-40-5	US 20090186879

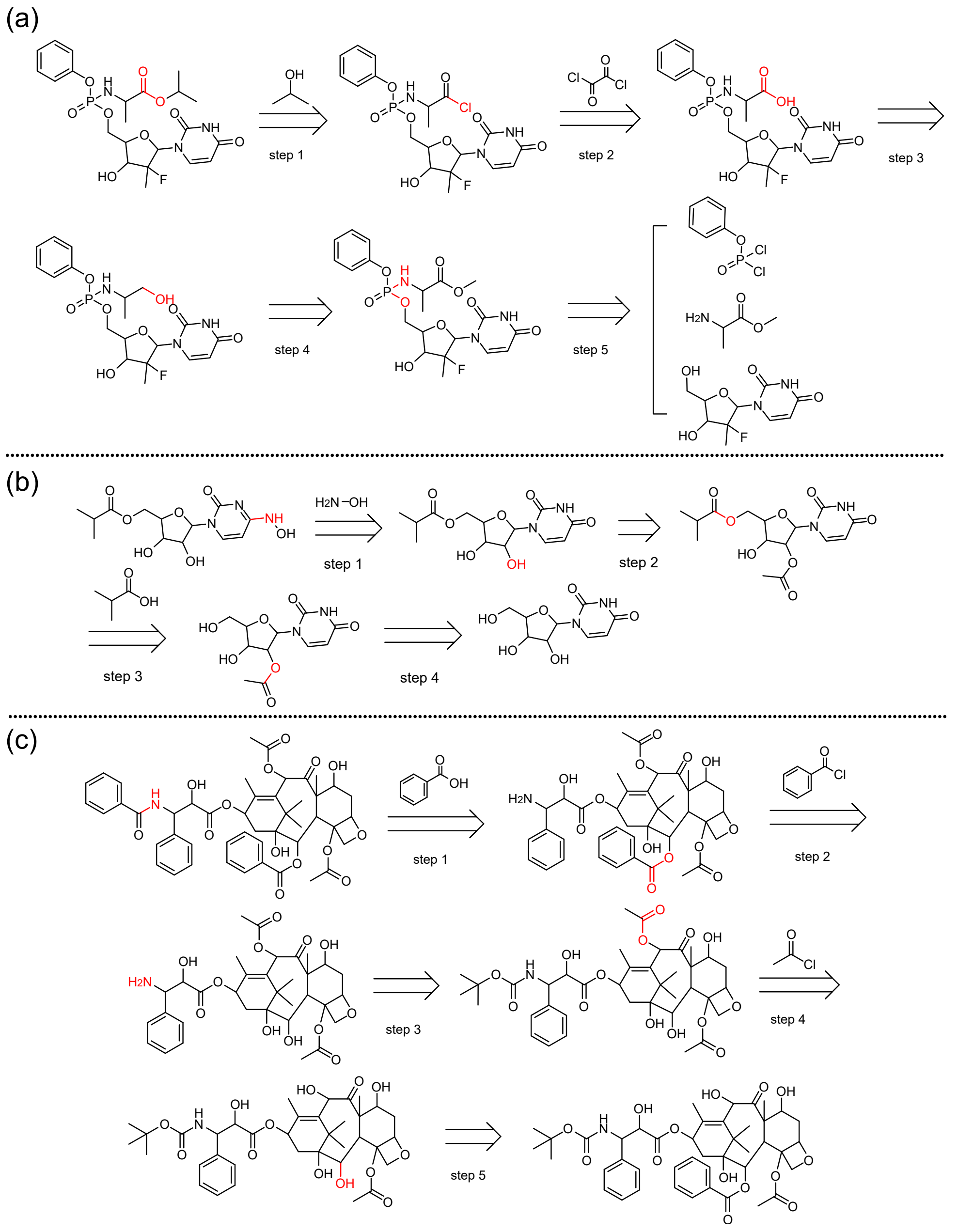

Supplementary Figure 1. The generated routes given by EG-MCTS for other three drug molecules, Sofosbuvir, Molnupiravir and Taxol. (a) The generated route given by EG-MCTS for Sofosbuvir, whose CAS number is 1190307-88-0. (b) The generated route given by EG-MCTS for Molnupiravir, whose CAS number is 2349386-89-4. (c) The generated route given by EG-MCTS for Taxol, whose CAS number is 33069-62-4. In experiment, we ignore their stereochemical structure. The molecules over the arrow are from $\mathcal{B}$. The atoms and bonds marked red are reaction center, which change in the reaction.
